# The mediating effects of self-leadership on perceived entrepreneurial orientation and innovative work behavior in the banking sector

**DOI:** 10.1186/s40064-016-3556-8

**Published:** 2016-10-21

**Authors:** Burcu Kör

**Affiliations:** Department of Management Information Systems, Boğaziçi University, Hisar Campus B Block, Bebek, 34342 Istanbul, Turkey

**Keywords:** Entrepreneurial orientation, Perceived entrepreneurial orientation, Self-leadership, Innovative work behavior, Innovative behavior, Banking sector

## Abstract

Innovative work behavior has been one of the essential attribute of high performing firms, and the roles of entrepreneurial orientation and self-leadership have been important for promoting innovative work behavior. This study advances research on innovative work behavior by examining the mediating role of self-leadership in the relationship between perceived entrepreneurial orientation and innovative work behavior. Structural equation modelling is employed to analyze data from a survey of 404 employees in banking sector. The results of reliability measures and confirmatory factor analysis strongly support the scale of the study. The results from an empirical survey study in the deposit banks reveal that participants’ perceptions about high levels of entrepreneurial orientation have a positive impact on innovative work behavior. The results also provide support for the full mediating role of self-leadership in the relationship between participants’ perceptions of entrepreneurial orientation and innovative work behavior. Additionally, this study provides some implications for practitioners in the banking sector to facilitate innovative work behavior through entrepreneurial orientation and self- leadership.

## Background

In recent years, the service sector has played an important role in promoting the growth of economy in many countries (Crevani et al. 2011; Wang and Tsai 2014). The role of the internet and web-based services and the growth in high-technology services indicate that knowledge-intensive business services are taking a more active economic role (Howells 2001; Desmarchelier et al. 2013). Within the knowledge-intensive business services, the banking sector contributes increasingly to the growth of the economy and economic activity (Jayawardhena and Foley 2000; Das 2013; Wang and Tsai 2014). In today’s global banking environment, innovation plays an extremely significant role for the competitive advantage (Liao et al. 2010; Bysted 2013). Scholars have also long recognized the crucial role of innovation in determining the competitive success of banking sector (Pennings and Harianto 1992; Prescott 1997; Mandeville 1998; Metcalfe and Miles 2012). Innovation gives banks and/or organizations a competitive advantage by increasing and sustaining a high performance, and by attracting new customers and retaining the existing ones (Cowling and Newman 1995; Gopalakrishnan and Damanpour 1997; Kör and Maden 2013; Rahman et al. 2015). De Jong and Den Hartog (2007) noted that organizations can become more innovative by taking advantage of their employees’ ability to innovate. In particular, innovation manifests itself through individuals’ innovative work behavior (Scott and Bruce 1994; De Jong and Den Hartog 2007; Pratoom and Savatsomboon 2012). Innovative work behavior (IWB) is of utmost importance for the organizations and/or banks to sustain innovation processes by including all behaviors regarding innovation (De Jong and Den Hartog 2007; Ojedokun 2012; Bysted 2013). IWB is at the base of high performance organizations through a broad set of behaviors: opportunity exploration, recognition of problem, transformation of ideas into tangible outcomes and strategically planning these outcomes integrated into organizational practice. However, few studies have focused on which individual and/or contextual factors affect individual innovation in the workplace. Hereby, it is crucial to find out what motivates or enables IWB (Scott and Bruce 1994; Carmeli et al. 2006; Pratoom and Savatsomboon 2012; Gomes et al. 2015b).

Entrepreneurial orientation (EO) is the presence of organizational-level entrepreneurship (Wiklund and Shepherd 2005). EO is mainly defined as strategic posture of a firm which is related to a firm-level strategy-making process that leads to innovativeness, leadership in the market or the ability to react fast and risk taking (Covin and Slevin 1989; Wiklund (1998, 1999); Rauch et al. 2009; Grimmer et al. 2013; Su and Sohn 2015). EO is also highly important in influencing willingness to innovate and in revealing the talents or behaviors of individuals that can sustain competitive advantage (Covin and Slevin 1988; Covin and Miles 1999). When organizations are simultaneously risk taking, innovative, and proactive with respect to their overall business operations, product offerings and technologies, and interactions with competitors, employees can perceive the work environment to have a high level of EO (i.e., the combination of innovativeness, risk taking and proactiveness). Perception of employees regarding the work environment that supports innovativeness, risk taking and proactiveness, can stimulate individual outcomes (Amo and Kolvereid 2005; Amo 2006).

Employees in all positions need to take more responsibility and make faster decisions in order to adapt to the modern business world that is in a constant change. Therefore, it is necessary to take steps toward dividing and/or sharing the leadership and giving place to self-directed groups or individuals. Within this framework, the concept of self-leadership (SL) has attracted the expanded attention of practitioners and scholars. SL behaviors involves the influence individuals exert over themselves to achieve the self-motivation and self-direction needed to behave in desirable ways (Manz 1986, 1992; Prussia et al. 1998; Houghton and Neck 2002). SL is defined as an individuals’ general combination of a systematic set of strategies through which individuals can control their own behavior and influence themselves towards achieving desired behaviors and outcomes (Prussia et al. 1998; Carmeli et al. 2006; D’Intino et al. 2007). The constellation of these strategies influences a perception of control, capability and responsibility which positively affects people’s behavior, effectiveness and performance outcomes (Manz 1983, 1992; Prussia et al. 1998). By utilizing the general combination of SL behaviors within organizations, innovative behavior can be triggered and deployed in the workplace (Carmeli et al. 2006; Kalyar 2011; Gomes et al. 2015b).

The ability to behave innovatively in the workplace is becoming more and more significant for continuous innovation in today’s business organizations (Boer and Gertsen 2003). As a result, researchers and practitioners are interested in identifying contextual and individual factors that affect innovative behavior (Scott and Bruce 1994; Ramamoorthy et al. 2005; Hammond et al. 2011; Romero and Martínez-Román 2012). The study of the effects of contextual and individual factors on IWB has been previously undertaken in the literature by a number of researchers (e.g., Scott and Bruce 1994; Ramamoorthy et al. 2005; Ng et al. 2010; Madrid et al. 2014; Ma Prieto and Pilar Perez-Santana 2014). Nevertheless, most of these studies have ignored or minimized the effects of individual perceptions of the work environment and leadership skills on innovative behavior (Pieterse et al. 2010; Hammond et al. 2011). Individual perceptions or cognitive interpretations of the work environment are referred to as psychological climate that provides a basis for behavior and affect (James and Sells 1981; Field and Abelson 1982). Accordingly, from the perspective of psychological climate theory, the current study investigates how individuals perceive a specific aspect of their work environment (i.e., perception of EO) and how this perception relates to SL skills and innovative behavior in the workplace. Despite the researchers’ interest in the effects of leadership skills on innovative behavior in the workplace, they tend to generally focus on transactional and/or transformational leadership skills (e.g., Basu and Green 1997; Janssen 2002; Renvers et al. 2008; Pieterse et al. 2010; Khan et al. 2012). The research on innovative behavior is insufficient in regards to the SL skills of the individuals in the workplace. Additionally, individuals are more likely to lead themselves in the workplace as well as to take risk in terms of generating and/or implementing ideas or trying something new, when they perceive high levels of EO (Amo and Kolvereid 2005; Amo 2006; Hammond et al. 2011). However, there is a lack of empirical evidence exploring the relationship between the perceived EO, SL and IWB. Empirical research on these dynamic relationships also needs to be expanded into different cultures, since majority of SL and IWB studies have been examined within the context of developed countries (Alves et al. 2006; Chen et al. 2012; Ugurluoglu et al. 2015). In addition, previous empirical researches on SL have neglected to specify the antecedents of SL and test the possible mediating role of SL, except the studies of Curral and Marques-Quinteiro (2009) and Pratoom and Savatsomboon (2012). In line with these arguments, this study provides an in-depth theoretical and empirical analysis about the relationship between EO, SL and IWB in a banking sector of a developing country. The context of this study is banking sector as this sector is forced to become more responsive to innovative demands because of infusion of information technology. To the best of the author’s knowledge, this is one of the first studies that theoretically specifies and empirically examines the relationship between the perception of EO, SL and IWB, and the mediating role of SL in the relationship between the perception of EO and IWB within the context of banking sector in a developing country. By doing so, the contribution of the study is not only to gain a deeper understanding of the relation between the perceived EO, SL and IWB but also to provide managers with guidance on how to facilitate individuals’ attitudes and/or perceptions to drive innovative behavior in the workplace. Given this context, the study also provides some suggestions for banks in developing countries to manage employees’ behavior or skills and accordingly maximize innovative behavior and SL skills. Both the individual and the organization can benefit reciprocally with the utilization of these behaviors and skills in the workplace.

The rest of this article is organized as follows: “Literature review and hypotheses development” section focuses on the key constructs in the study, which are EO, SL and IWB, and develops the rationale for the conceptual model and hypotheses. Subsequently, the method and the results are presented. The last section reveals the discussion and conclusion as well as the limitations and future research suggestions.

## Literature review and hypotheses development

### EO and IWB

EO provides a competitive advantage by efficiently regulating the processes and strategies, and by discovering the talents or behaviors in the organizations. In this sense, EO is highly important for environmental opportunities and/or their benefits: creating a dynamic, flexible, innovative and competitive organizational structure that is especially successful for shaping the work-environment, and for reaching the advantage and long term gains (Zahra 1986; Covin and Miles 1999).

EO contains entrepreneurial behaviors in the processes and methods applied by the organizations, as well as the strategies for discovering and benefiting from existing or potential opportunities leading to a maximum gain (Rauch et al. 2009; Tang et al. 2009; Wales et al. 2013). Wiklund and Shepherd (2005) defined EO as ‘a firm’s strategic orientation, capturing specific entrepreneurial aspects of decision-making styles, methods, and practices’ (p. 74) and described EO as having dimensions of innovativeness, proactiveness and risk-taking.

Innovativeness is defined as ‘willingness to support creativity and experimentation in introducing new products/services, and novelty, technological leadership and R&D in developing new processes’ (Lumpkin and Dess 2001, p. 431). Proactiveness refers to a posture of anticipating future demands, a firm’s future looking perspective, and an actively seeking opportunities and/or novel ways to create change and to shape the environment, thereby introducing new products, processes and/or services ahead of the competition (Covin and Slevin 1986; Lumpkin and Dess 2001; Fuentes-Fuentes et al. 2015; Su and Sohn 2015). Risk-taking is defined as the willingness of an organization to support projects where the outcomes are unknown, devoting resources and capital to projects for the chance of desirable outcomes, and entering new markets which can be highly profitable in the long run (Lumpkin and Dess 2001; Antoncic and Hisrich 2003; Wiklund and Shepherd 2005). Although innovativeness, risk taking, and proactiveness are important dimensions that entrepreneurial firms may exhibit, these three dimensions of EO act together to comprise an entrepreneurial firm’s basic strategic orientation (Miller 1983; Covin and Slevin 1989). Covin and Slevin (1988) argued that EO of an organization could be best measured by summing together the three dimensions, and so these dimensions should be aggregated together when conducting research in the field of entrepreneurship (Covin and Slevin 1989; Naman and Slevin 1993; Wiklund 1999). In line with view of Covin and Slevin (1988, 1989), EO is treated as a unitary concept in order to assess the overall level of a firm’s EO. In this light, the aggregated EO construct provides re-regulation of processes in organizations in the rapidly changing circumstances and exposure of either competitive advantage or innovative behaviors in the workplace (Wiklund and Shepherd 2005; Amo 2006).

In the rapidly changing circumstances, products have shorter lifespan which affects their innovation processes. Due to this, organizations need to develop new products, services and processes more frequently or shorten the duration between innovations. Generation of ideas and conversion of those ideas into lucrative and beneficial products, as well as services for organizations is significant (Han et al. 1998). Employees’ IWB can facilitate innovation in the workplace by integrating employees within the development and implementation of innovation processes (Reuvers et al. 2008; Noefer et al. 2009; Pratoom and Savatsomboon 2012). IWB is defined as ‘all employee behavior directed at the generation, introduction and/or application (within a role, group or organization) of ideas, processes, products or procedures, new to the relevant unit of adoption that supposedly significantly benefit the relevant unit of adoption’ (De Spiegelaere et al. 2012, p.7). Scott and Bruce (1994) state that IWB is a multi-layered process, and it issues all the aspects of innovation processes which fundamentally include creativity and application stages. According to De Jong and Den Hartog (2010), IWB contains all the processes of idea exploration, generation of ideas that are completely new or adapted, seeking out support for the ideas and implementation of the ideas. In line with the view of De Jong and Den Hartog (2010), IWB comprises four inter-related sets of behavioral activities, namely (1) idea exploration, (2) idea generation, (3) idea championing, and (4) idea implementation.

Innovative behavior generally starts with idea exploration from the realization of opportunities and problems that need to be solved. Idea exploration includes searching for new methods or thinking about alternative methods to develop existing products, services or processes (Ong et al. 2003; De Jong and Den Hartog 2007, 2010). Idea generation is generally about development of new solutions and original or innovative ideas for problems, obscurities or tough matters. Likewise, employees find new approaches, ways or ideas while fulfilling their responsibilities and while seeking out new ideas for their present work methods, techniques or tools in the stage of idea generation (Janssen 2000; Kleysen and Street 2001; Dorenbosch et al. 2005). The generation of innovative ideas in organizations is not adequate to transform those ideas into tangible outcomes. In order to put the ideas into effect, building support and coalitions for those ideas is necessary (Kleysen and Street 2001; De Jong and Den Hartog 2010). Idea championing is expressed as the concept of mobilizing support, persuading the other employees and motivating important organizational members for innovative ideas (Dorenbosch et al. 2005; De Jong and Den Hartog 2010; Madrid et al. 2014). Persuasion of employees in organizations about the importance of generated ideas has a notable effect in increasing the chance of putting them into practice. Therefore, mobilizing support and receiving approval for the innovative ideas, increase employees’ participation in the innovation processes while they are significant to processes in disseminating innovative behavior within the organization (Dorenbosch et al. 2005; Carmeli et al. 2006; Reuvers et al. 2008). The final process of IWB involves idea implementation which is indicated as transformation of innovative ideas into tangible outcomes. Idea implementation encompasses the development of new products, services or processes and the implementation of the idea within the organization (De Jong and Den Hartog 2010; Messmann and Mulder 2011; Caniëls et al. 2014).

When organizations construct an environment that supports EO, they can form a proper ground, not only for organizations, but also for individuals to attain their goals (Wiklund and Shepherd 2003; Altinay and Altinay 2004; Rauch et al. 2009). EO can create policies, processes and strategies to be helpful in adoption of proactive posture, willingness to support innovation and creativity processes, as well as willingness to accept risks as necessary antecedents for the organizations’ competitive advantages, while promoting individuals’ innovative behavior in the workplace (Covin and Slevin 1988; Covin and Miles 1999; Mumford et al. 2002; Amo 2006). In such a work environment, bureaucracy, complex processes, hierarchy and the elements preventing creativity and/or innovative behaviors can be removed or reduced (Thornberry 2001; Yuan and Woodman 2010). When individuals perceive such a work environment (i.e., encourages innovativeness, has inclination to act proactively or is safe for risk taking), individuals are more likely to proceed with innovative behavior in the workplace (Amo and Kolvereid 2005; Amo 2006; Yuan and Woodman 2010; Hammond et al. 2011). Employees’ perceptions regarding the overall level of a firm’s EO, could trigger and deploy employees’ IWB. However, there is a lack of empirical evidence exploring the relationship between perceived EO and IWB. In line with these arguments, it is hypothesized that:

**Hypothesis 1**: Perceived EO is positively related to IWB.

### SL and its relationship with EO and IWB

Under the global conditions, traditional leadership roles undergo changes. Rather than effects of leaders on followers, SL of each individual in an organization can help to maximize the contributions of individuals to the organization. Organizations in today’s conditions expect more creativity, innovation, quick and flexible actions, collaboration, and initiation in the rapidly changing conditions of their employees. They also expect their employees to exhibit and develop their leadership abilities. In this framework, not only the managers or leaders of today’s organizations, but also employees are required to affect themselves, establish their self-management and have the ability of making decisions, and so SL proves to be quite important (Pearce 2007; Bryant and Kazan 2012; Houghton et al. 2014).

SL has a broad spectrum of theoretical origins. SL operates within the framework of self-regulation, social cognitive, motivation, self-efficacy, self-management and self-influence theories; and integrates these theories in a complete set of behavioral and cognitive strategies (Manz 1986; Houghton and Neck 2002; Neck and Houghton 2006). SL provides the enhancement of personal effectiveness through specific sets of behavioral and cognitive strategies (Houghton and Neck 2002; Neck and Houghton 2006; Andressen et al. 2012).

According to Manz (1986), SL is ‘a comprehensive self-influence perspective that concerns leading oneself toward performance of naturally motivating tasks as well as managing oneself to do work that must be done but is not naturally motivating’ (p. 589). SL is defined as a set of strategies that address what is to be done (e.g., standards and objectives) and why (e.g., strategic analysis) as well as how it is to be done (Manz 1991). SL strategies may be divided into three general categories: behavior-focused strategies, natural reward strategies and constructive thought pattern strategies (Houghton and Neck 2002; Neck and Houghton 2006).

Behavior-focused strategies encompass the processes in which individuals affect themselves and direct their own behaviors through SL in order to be encouraged in enjoyable behaviors and to manage their necessary tasks when exposed to unpleasant behaviors (Bligh et al. 2006). The behavior-focused strategies include self-observation, self-goal setting, self-reward, self-punishment and self-cueing. Self-observation helps to gather systematic information regarding individuals’ behaviors, thoughts or emotions and to follow self-development. Self-observation also detects behaviors desired to be increased or reduced and to improve self-awareness about the reasons of those behaviors. Therefore, individuals can efficiently manage or evaluate themselves and take actions to remove or change negative behaviors (Manz 1980). Self-goal setting includes the capability of setting goals for the things that individuals wish to succeed in the future, for themselves and for their performance. The ability to set goals plays a prominent role for individuals in determining their priorities or their own way, developing motivation, self-leading and fulfilling their responsibilities. Self-reward is related to individual reward with the things pleasant to the individual. Self-reward provides individuals with motivation to reach the desired behaviors and goals or to successfully fulfill a task (Bryant and Kazan 2012). Self-punishment pertains to individuals’ own evaluation and correction of themselves. Self-punishment includes self-criticism, self-evaluation or self-punishment in order to correct himself/herself in the situations of weak or bad performance, inefficacy and failure during a task. As opposed to self-reward, self-punishment aims to remove the undesired behaviors of individuals (Manz 1980, 1986; Neck and Houghton 2006). Self-cueing contains the role models taken as examples and objects determined by individuals in the course of realizing goals or fulfilling necessities. Thereby, self-cueing can be considered as stimulants, and it helps individuals to focus their attention on the tasks (Manz 1991; Neck and Houghton 2006; Bryant and Kazan 2012). Accordingly, behavior-focused SL strategies are deployed to reduce or remove behaviors that can cause failure or unwanted situations and to encourage desired behaviors that can bring about successful consequences (Bligh et al. 2006).

Natural reward strategies are based on the approach that highlights the positive aspects of a task to be done. Natural reward strategies bring along an internal motivation increase, especially when individuals deal with various problems. Individuals try to tackle the problems by creating motivating situations instead of ignoring those problems while using this strategy (Houghton and Neck 2002; Amundsen and Martinsen 2015). In natural reward strategies, there are two approaches helping to increase the efficacy of SL: the first of those approaches is about the acts of an individual such as making the task or work environment more enjoyable or as focusing on the pleasant aspects of his/her job. The second approach in natural reward, is about ‘shaping perceptions by focusing attention away from the unpleasant aspects of a task and refocusing it on the task’s inherently rewarding aspects’ (Neck and Houghton 2006, p. 272).To summarize, natural reward strategies can affect individual’s eagerness and competence to work positively and can help to enhance his/her motivation up to high levels.

Constructive thought pattern strategies involve the development of new thoughts or thought-patterns and make a habit out of them which would influence individual’s performance positively (Anderson and Prussia 1997; Houghton and Neck 2002; Neck and Houghton 2006). Constructive thought pattern strategies include visualizing successful performance, self-talk and evaluating beliefs and assumptions. Visualizing successful performance is the cognitive imagination in the mind of the individual before facing the situation. Visualizing successful performance increases the possibility of fulfilling the task effectively due to the mental rehearsal prior to the task (Houghton and Neck 2002). Self-talk is defined as the quiet talk of the individual with him/herself or as the internal talk, and it involves mental self-evaluations and reactions (Houghton and Neck 2002; Neck and Houghton 2006). Evaluating beliefs and assumptions include the evaluation of habits, thinking methods or models that are developed by individuals. According to Ho and Nesbit (2009), evaluating beliefs and assumptions concerns ‘examining one’s thoughts, especially self-defeating thoughts that detract from successful task performance’ (p. 454). Evaluating beliefs and assumptions aims to help one to eliminate undesirable and dysfunctional habits (Houghton and Neck 2002; Ho and Nesbit 2009). Consequently, it is made possible by constructive thought pattern strategies that individuals use their experiences and/or thoughts positively and develop the desired behaviors (Houghton and Neck 2002; D’Intino et al. 2007). And so, SL strategies improve individual effectiveness in the organizations (DiLiello and Houghton 2006).

The extant literature concerning SL includes much that displays a high-level description of behaviors or characteristics, and possible outcomes; however, little is known about the forces behind these behaviors and/or characteristics in a work setting. Renn and Huning (2008 as cited in Şahin 2011) contended that SL skills may be dependent upon how the individuals perceive their work environment. As indicated previously, individuals’ perceptions of their work environment is conceptualized as psychological climate (Parker et al. 2003). From this perspective, Renn and Huning (2008) as cited in Şahin (2011) examined how psychological climate can explain “the essential features believed to influence the quality of SL” in the workplace (p. 4). Employees’ perceptions regarding the work environment may have a significant impact on employees’ work attitudes and behaviors (Parker et al. 2003; Şahin 2011) and how employees lead themselves effectively in the workplace (Renn and Huning 2008). According to Renn and Huning (2008 as cited in Şahin 2011) psychological climate for SL defined as “perceptions of the events, practices, procedures, and behaviors that management rewards, supports, and expects with respect to SL” (p.5). Drawing on psychological climate theory, it is suggested that perceived EO sends an implicit message to employees regarding the overall perceptions of organizational support for innovation, openness to change, acting proactively and risk taking, which in turn would provide freedom, independence, autonomy or more active role for employees to make decisions or participate in decision making, as well as reduce bureaucracy to act quickly and transmit greater confidence and self-esteem in the workplace (Roberts and Foti 1998; Yun et al. 2006). When employees perceive such a work environment that encompasses organizational attributes such as openness to change, autonomy and support for risk taking, they can learn how to set their own goals, how to influence themselves and how to take autonomous action, as well as how to lead themselves (Norris 2008; Kalyar 2011; Eliason 2013). Additionally, in the organizations where SL is supported, every employee proves efficiency in sorting out problems that are related to both themselves and the organization, as well as taking responsibilities for their work and themselves (Pearce and Manz 2005; Bryant and Kazan 2012; Eliason 2013). In this respect, it is assumed herein that SL skills can be thought of as being influenced by perceived EO. However, the lack of research in the relationship between perceived EO and SL confines understanding of exactly how perceived EO is affecting SL. Based on these arguments, the following hypothesis is postulated:

**Hypothesis 2**: Perceived EO is positively related to SL.

Based on the intensive literature search, the common theme in improving effective leadership is starting with knowing and managing oneself (Bennis 1994; Drucker 1999; Yukl 2001; Boyatzis and McKee 2013). In this context, SL is increasingly gaining importance. This is because SL is a process of self-influence to achieve an optimum state of motivation, as well as self-discovery, self-regulation and self-direction that give strength, purpose, meaning and direction to the effort toward effectiveness during task performance (Manz 1986; Neck and Manz 1992; Manz and Sims 2001; Stewart et al. 2011). According to Manz (1986) and Unsworth and Mason (2012), the combination of SL strategies is likely to improve performance above and beyond the individual strategies alone, as well as helps individuals to maximize personal and professional strengths and minimize personal and professional weaknesses. Furthermore, SL literature has suggested a number of predictable outcomes, which may serve as the mechanisms that affect individual, group and organizational effectiveness and performance (Neck and Houghton 2006; DiLiello and Houghton 2006). Several scholars suggest that SL skills are essential to organizations that need continuous innovation (Pearce and Manz 2005; DiLiello and Houghton 2006; Neck and Houghton 2006). Because of the changing nature of work, employees at all levels of the organization should participate in innovation activities and demonstrate higher levels of self-confidence about performing these activities (Thatcher and Perrewe 2002; Ong et al. 2003; Wu et al. 2014). SL skills provide employees with a general combination of behavior-focused, natural reward and constructive thought pattern strategies that employees can learn and implement in a wide range of environments, thus giving them psychological resources and self-confidence that strengthens their positive affect resources, which in turn positively influences their subsequent outcomes (Neck and Houghton 2006; Carmeli et al. 2006; Gomes et al. 2015a). Within this context, employees need to be able to lead themselves to behave innovatively in the workplace (Carmeli et al. 2006; Pratoom and Savatsomboon 2012; Gomes et al. 2015a, b). Few studies examined how the combination of SL skills influence IWB (e.g., Carmeli et al. 2006; Curral and Marques-Quinteiro 2009; Kalyar 2011; Pratoom and Savatsomboon 2012; Gomes et al. 2015a). Within these studies, all but Pratoom and Savatsomboon (2012) support the hypothesis that the combination of SL skills directly affect IWB. Therefore, research in this area is still in nascent stage (Carmeli et al. 2006; Pratoom and Savatsomboon 2012; Gomes et al. 2015b). Building on SL theory, which conceptualizes the combination of SL skills as a determinant of the innovative behavior, and empirical findings (Carmeli et al. 2006; Curral and Marques-Quinteiro 2009; Kalyar 2011; Gomes et al. 2015a), the following hypothesis is formulated:

**Hypothesis 3**: SL is positively related to IWB.

### Potential mediating effects of SL

EO reflects a tendency to engage in and/or support innovation, risk taking and proactiveness, which in turn creates an appropriate culture, climate and/or structures for innovative behavior and innovation processes (Rauch et al. 2009; Wales et al. 2013). However, innovation processes are characterized by certain levels of uncertainty and complexity. Under these conditions individuals are the crucial actors in the innovation process, therefore they should have certain level of internal force to face the uncertainty, complexity and resistance in innovation (Carmeli et al. 2006; Kalyar 2011). This internal force is rooted in SL skills (Carmeli et al. 2006; Pratoom and Savatsomboon 2012). SL represents a combination of behaviors, attitudes, and cognitions which are a competence for leading oneself across challenging and performing situations (Prussia et al. 1998; Houghton and Neck 2002). Self-leaders are more likely to view themselves as capable to perform at a higher level (Neck and Manz 2012), as well as exert over themselves to achieve the self-motivation and self-direction needed to behave in desirable ways (Manz 1992). According to Norris (2008), in environments where employees perceive the encouragement of leading themselves, SL skills may be useful for maximizing personal and professional strengths and performance. Pratoom and Savatsomboon (2012) also proposed that when the group culture encourages risk taking, values innovation and supports learning by trial, SL and intrinsic motivation of individual would be positively affected, which in turn would foster individual innovation. In this respect, perceptions of employees regarding the work environment that support a constellation of innovativeness, risk taking and proactiveness, may encourage employees who possess SL skills to display innovative behavior. When such a work environment is perceived by employees, SL skills may contribute to the conversion of IWB into a daily process, either to make habits out of the desired innovative behaviors in the workplace or to remove elements that prevent individuals’ IWB, owing to the fact that SL skills provide self-efficacy, internal motivation, self-influence and self-awareness (Neck and Manz 1996; Houghton and Jinkerson 2007). It may therefore be presumed that perception of EO influences the utilization of general SL skills which subsequently affects IWB. Examining the mediating role of SL would be added to our understanding about the nature of relationships among the perceived EO, SL and IWB. Accordingly, the following hypothesis is formulated:

**Hypothesis 4**: SL mediates the relationship between perceived EO and IWB.

There is a growing body of literature about SL and its importance in the workplace, but very few studies have adequately examined the mediating role of SL. For example, in Curral and Marques-Quinteiro’s (2009) research, mediation analysis supports the hypotheses that SL skills fully mediated the relationship between learning goal orientation and work role innovation and partially mediated the relationship between intrinsic motivation and work role innovation. The other study found that SL didn’t mediate between group culture and group members’ innovation (Pratoom and Savatsomboon 2012). Additionally, to the best of the author’s knowledge, there has been no research in the literature regarding SL as a full or partial mediator of the relationship between perceived EO and IWB. Baron and Kenny (1986) indicated that partial mediation is the most frequent model in psychology research. Thus, the partial mediation model is the practical choice, if theory and research are ambiguous on form of mediation effect. However, according to James et al. (2006), if theory and research are insufficient to hypothesize complete or partial mediation, testing for full mediation is recommended since full mediation model is the most parsimonious mediation model. James et al. (2006) also indicated that full mediation should serve as the focal or baseline model in evaluating mediation. In the current study, perceived EO has also been proposed to influence IWB through its’ effect on the utilization of general SL skills. Therefore, the proposed hypothesis was tested with a full mediation and compared with a partial mediation model that included the possible direct effect among the main constructs.

## Methods

### Data collection

The data was collected between June, 2014 and November, 2014. The questionnaire consisted of 61 items divided among topics: EO, SL and IWB, and questions about participants’ demographic characteristics. Additionally, the data was collected from the different work units of deposit banks operating in Istanbul, Turkey. According to statistical reports of The Banks Association of Turkey (2014), as of June 2014, the number of deposit banks (privately-owned banks, state-owned banks and foreign deposit banks founded in Turkey) operating in Turkey was 26 of which 11 privately-owned, 3 state-owned and 12 foreign deposit banks. The dataset for this study was compiled from 17 banks among these deposit banks: 8 privately-owned, 3 state-owned and 6 foreign deposit banks. These banks have higher number of branch offices and employees than the banks in other groups and unreached deposit banks. In addition, the banks in the sample represent the general classification of deposit banks in Turkish banking system. The participants of the study were randomly selected from the positions of associate, manager and senior manager within these banks who were knowledgeable about the key constructs of the study. Multiple participants were also selected from each bank. Participants in this study answered the questionnaire in a voluntary manner and were informed of the aim of the survey. Participants were also assured of the anonymity and the confidentiality of their answers. Questionnaires were administered to 461 employees and a total of 404 (88 % respond rate) were usable. The demographic characteristics of the respondents are represented in Table 1. The majority of the respondents had a college degree (76 %), followed by post-graduate (18.8 %) and high school degrees (5.2 %).

**Table 1:** Demographic characteristics of the respondents

	Respondents (n = 404)	
	No.	%
Gender		
Male	177	43.8
Female	227	56.2
Age		
20–30	152	37.6
31–40	189	46.8
>40	63	15.6
Education		
High-school	21	5.2
Undergraduate	307	76.0
Postgraduate	76	18.8
Work experience		
1–5	141	34.9
6–10	102	25.2
>10	161	39.9
Job tenure		
1–5	212	52.5
6–10	98	24.3
>10	94	23.3
Position/title		
Associate	93	23.0
Manager	115	28.5
Senior manager	196	48.5

N = 404

### Measures

All items in the questionnaire were measured on a five-point Likert scale ranging from ‘strongly disagree’ to ‘strongly agree’. The questionnaire was originally developed in English, and then underwent a back-translation procedure (Bhalla and Lin 1987). Once the translation process was finalized, the content validity, clarity and accuracy of the questionnaires were checked and approved by two faculty members, three doctoral students and two managers from deposit banks. All correlational analyses, ANOVA analyses, independent t test, tests of reliability, confirmatory factor analyses, statistical techniques of common method variance and structural equations modelling (SEM) analysis were performed by using the software programs SPSS (Version 22.0) and AMOS.

#### Entrepreneurial orientation

EO was measured by nine items developed by Covin and Slevin (1989), based on the work of Miller and Friesen (1982), and Khandwalla (1977). Covin and Slevin’s scale is one of the widely used measures of EO that have been utilized by several scholars (e.g., Wiklund and Shepherd 2003; Swierczek and Quang 2004; Wales et al. 2013).

#### Self-leadership

SL was assessed using a version of the revised self-leadership questionnaire (RSLQ). The RSLQ consisted of 35 items and was developed by Houghton and Neck (2002) and based on Anderson and Prussia’s (1997) self-leadership questionnaire and Cox’s (1993) unpublished SL scale. The RSLQ confirmed to be an effective measure of SL and was found to have a good reliability and validity across a number of empirical studies (e.g., Houghton et al. 2004; Houghton and Jinkerson 2007; Doğan and Şahin 2008; Curral and Marques-Quinteiro 2009; Şahin 2011; Tabak et al. 2013). An analysis of reliability on the RSLQ items resulted in a high corrected item-total correlation of more than .3 was found for all the items except one (referring to: I tend to get down on myself in my mind…). This item was excluded from further analysis.

#### Innovative work behavior

To measure the IWB, the scale of De Jong and Den Hartog (2010) was used. The scale consisted of 17 items and derived from Scott and Bruce (1994), Janssen (2000) and Kleysen and Street (2001). Self-reported data was used. This is in line with Janssen’s (2000) suggestion that ‘a worker’s cognitive representation and reports of his or her own IWB may be more subtle than those of his or her supervisor, since a worker has much more information about the historical, contextual, intentional and other backgrounds of his or her own work activities’ (p. 292).

#### Control variables

To control the existence of confounding variables from demographic characteristics on the relationship between the predictors and outcome variables, position (1 = associate, 2 = manager, 3 = senior manager) and gender (0 = male, 1 = female) were applied as covariates suggested by prior research (Janssen 2000, 2004; Carmeli et al. 2006). Position was controlled because it can impact individual’s ability and/or behavior to promote IWB. Gender was applied as covariates because some researches indicate that there are differences between the male and female in terms of IWB (Janssen 2000; Carmeli and Spreitzer 2009), while others indicate that there are no differences (Carmeli et al. 2006; Reuvers et al. 2008; Pratoom and Savatsomboon 2012).

Confirmatory factor analysis was performed to assess the validity of the multi-item measurement scale. According to Hair et al. (2009), comparative fit index (CFI) values above .90 were usually associated with a model that fits well. The cutoff value of .05 or less should be used for the root mean square error of approximation (RMSEA). In general, if the ratio between the Chi square goodness-of-fit measure and degrees of freedom was less than two, the model was accepted (Hair et al. 2009; Tabachnick and Fidell 2001). Hu and Bentler (1999) suggested that standard root mean square residual (SRMR) should be less than .08. In accordance with the cutoff points of these fit indices, the measurement model results indicate a good fit to the data (χ^2^/df  =  1.71, RMSEA  = .042, CFI  =  .923, RMR = .04, SRMR  =  .05).

Table 2 provides information about the Cronbach’s α, factor loadings and composite reliability. Internal consistency was assessed for each constructs using Cronbach’s α. Cronbach’s α ranges from .848 to .948 (corrected item-total correlation > .3), which indicates that all constructs have acceptable reliability. Factor loadings are above the recommended value of .30 (for sample size 350 or greater), and all factor loadings were significant (Hair et al. 2009). The composite reliability values ranged between 0.836 and 0.948, which exceeded the recommended .70 threshold value; therefore construct reliability can be assumed (Bagozzi and Yi 1988).

**Table 2:** Factor loadings, Cronbach’s α and composite reliability

	Factor loadings	Cronbach’s α	Composite reliability
IWB	.587–.800	.948	.948
Perceived EO	.627–.852	.848	.836
SL	.609–.906	.932	.946

### Common method variance

The data for this study were collected using the self-report questionnaire that may lead common method bias or variance. According to Podsakoff et al. (2003), several procedural and statistical techniques should help to minimize potential problems for common method variance (CMV): first, assuring anonymity and confidentiality to all participants; second, using reverse code items in the questionnaire to reduce the potential effects of response pattern; third, highlighting the value of the research for the participants’ firms; and forth collecting data from participants who had knowledge about their firms. In addition, Harmon’s single-factor test for CMV was performed (Podsakoff et al. 2003). The results of the Harmon’s single-factor test show that more than one factor had an eigenvalue greater than 1, and first factor accounted for 30.15 % of the total variance explained (65.72 %). As Table 3 shows, the highest correlation among the principal constructs is .60, far less than the problematic level of CMV (e.g., .90) (Bagozzi et al. 1991). Furthermore, a latent CMV factor was included in the measurement model, and the loadings on this method factor were statistically insignificant, as well as the relationship between the variables were not affected by the CMV factor (Podsakoff et al. 2003). The results of these tests suggest that CMV is likely not a serious concern in the present study.

**Table 3:** Means, standard deviations and correlations

	M	SD	1	2	3	4	5
Gender	.56	.49	–				
Position	2.26	.80	.038	–			
IWB	4.08	.75	.081	.060	–		
Perceived EO	3.41	1.0	.081	.036	.21**	–	
SL	3.92	.88	.112*	.085	.60**	.33**	–

Gender is coded 0 = male, 1 = female

* p < 0.05; ** p < 0.01; *** p < 0.001

## Results

Table 3 reports correlations and descriptive statistics for all variables. As indicated in Table 3, IWB was positively associated with both EO (r = .21, p < .001) and SL (r = .60, p < .001), and EO was positively correlated with SL (r = .33, p < .001). These results were consistent with the theoretical predictions and they provided initial support for the hypotheses of the study.

Independent-samples t-tests were conducted to compare EO, SL and IWB for gender differences. These tests indicated that female (mean ± SD: 4 ± .49) are more likely than male (mean ± SD: 3.8 ± .53) to use general SL strategies (t = 2.471, p = .014) and differences between means were non-significant for EO and IWB (t = 1.679, p = .094; t = 1.603, p = .110, respectively). Additionally, ANOVA tests were conducted to control for mean difference due to the position of participants. ANOVA showed no differences between positions regarding scores of EO, SL and IWB (F_(2, 401)_ = .273, p = .761; F_(2, 401)_ = 1.476,  p = .230; F_(2, 401)_ = .692, p = .501, respectively).

This study applies SEM to verify the hypotheses, and utilizes AMOS software to obtain the empirical results by means of the method of maximum likelihood estimation (MLE). The first step in SEM is to assess the overall model fit with some fit indices (Fang et al. 2014). The overall goodness of fit indices indicates that the hypothesized models are good representations of the structures underlying the data (Baumgartner and Homburg 1996). The overall fit measures of the full model in the SEM indicates that the fit of the model is acceptable (χ^2^/df  =  1.706, RMR = .042, RMSEA = .042, SRMR = .05, IFI = .924, CFI = .923).

Table 4 shows the findings, which incorporate the paths, betas, significance levels and results. The findings illustrated that EO was positively associated with IWB (β = .236, p < .001); therefore, Hypothesis 1 was supported. The results also showed that EO was positively associated with SL (β = .364, p < .001); therefore, Hypothesis 2 was supported. In addition, the results demonstrated that SL was positively associated with IWB (β = .633, p < .001); therefore, Hypothesis 3 was also supported (see Table 4).

**Table 4:** The results of hypotheses testing

Paths	Betas	Hypotheses	Results
Perceived EO → IWB	.236***	H1	Supported
Perceived E → SL	.364***	H2	Supported
SL → IWB	.633***	H3	Supported

* p < 0.05; ** p < 0.01; *** p < 0.001; all coefficients are standardized

Hypothesis 4 predicts that SL mediates the relationship between EO and IWB. SEM was used to test the relationship between antecedent or predictor (perceived EO), mediator (SL) and outcome (IWB) simultaneously, since SEM has the advantages of correcting for unreliability of measures (MacKinnon et al. 2007; MacKinnon 2008). As noted above, prior research and theory do not provide a compelling rationale for whether SL will partially or fully mediate the relationship between perceived EO and IWB. Hence, in line with the James et al. (2006) recommendation (i.e., full mediation represents the best choice of a baseline model), the proposed hypothesis was tested with a full mediation model and then compared with a partial mediation model.

Table 5 provides the comparative data for the null, partial versus full mediation models. The null or nonmediated model considers the direct effect of the independent or antecedent variables (perceived EO) on the dependent or outcome variable (IWB). In the partial mediation model, the antecedent influences the outcome variable both directly and indirectly through its effect on the mediator (SL). In the full mediation model, the antecedent only influences the outcome indirectly through its effect on the mediator. As shown in Table 5, results indicated that null model represented an acceptable fit to data, except SRMR statistic: χ^2^/df  =  1.831, RMR = .115, RMSEA = .045, SRMR = .1709, IFI = .910, CFI = .909. The null model had significantly worse fit than the partial mediation model: χ^2^ difference (df = 2) = 202.368, p < .001. Fit indices also indicated that full mediation model provided a good fit to data: χ^2^/df  =  1.705, RMR = .042, RMSEA = .042, SRMR = .0538, IFI = .924, CFI = .923, as did the partial mediation model: χ^2^/df  =  1.706, RMR = .042, RMSEA = .042, SRMR = .0537, IFI = .924, CFI = .923. However, the alternative model of partial mediation effect of SL did not provide a better fit to data than the full mediation model and the fit indices remained almost unchanged: χ^2^ difference (df = 1) = .036, n.s. Thus, the rule of parsimony indicates the fully mediated model is the preferred model (James et al. 2006). In the full mediation model, perceived EO had a significant effect on SL (β = .365, p < 0.01). SL in turn exerted a significant effect on IWB (β = .634). All of the regression coefficients were significant and in the expected direction. Therefore, full mediation model was supported.

**Table 5:** Fit indices for covariance structure analyses

Model	χ^2^	df	χ^2^/df	RMR	RMSEA	SRMR	IFI	CFI	χ^2^ difference
1. Null	2918.546	1594	1.831	.115	.045	.1709	.910	.909	
2. Partial mediation	2716.178	1592	1.706	.042	.042	.0537	.924	.923	202.368^a^
3. Full mediation	2716.214	1593	1.705	.042	.042	.0538	.924	.923	0.849^b^ (ns)

*ns* not significant

^a^Model 2–1 difference

^b^Model 3–2 difference

Further support for the Hypothesis 4, Sobel’s formula was used to test the fully mediating role of SL in the relation between perceived EO and IWB. Sobel’s test confirmed that SL fully mediated the relation between perceived EO and IWB (z = 4.406, p < .001). Furthermore, a resampling method known as bootstrapping was used to test the significance of the mediational effect of SL in the relation between perceived EO and IWB. The bootstrap method is considered a more rigorous approach and has greater statistical power than a majority of other procedures (MacKinnon et al. 2004; Morrow et al. 2008). For this study, the bootstrap process was generated 1000 random samples from the dataset to construct a 95 % standardized confidence interval. Results indicated that the mediational effect of SL in the relation between perceived EO and IWB was significant (lower bound = .158; upper bound = .303; p < .01). These findings supported the hypothesis that SL fully mediates the relationship between EO and IWB. Thus, in sum, Hypothesis 4 was again supported.

## Discussion and conclusion

Emergence of innovation behavior in the workplace is a critical factor in helping organizations to gain competitive advantage. Surprisingly, few empirical studies focus on what motivates or enables innovative behavior in the workplace. This study points to a gap in the literature about individual-level and organizational-level variables that could affect IWB, and furthermore this study makes valuable contributions to the growing body of literature on EO, SL and IWB by examining the effects of EO on IWB through SL.

Individuals’ perceptions of entrepreneurial activities (e.g., innovativeness, proactiveness and risk taking) facilitate empowerment, thinking ‘outside of the box’, coping with uncertainty and/or complexity associated with the innovation process without fear of punishment or failure, and proactively participating in the innovation processes, thereby exhibiting IWB (Amo 2006; Yuan and Woodman 2010). In agreement with the findings of Amo (2006), results of the study indicate that individuals are more likely to engage in innovative behavior when firms have high levels of EO. This study also shows empirical evidence exploring the relationship between perceived EO and SL. In organizations with strong EO climate, processes or practices are designed to create environments where innovativeness, proactiveness and risk taking behaviors stimulate SL skills of employees. Additionally, at the practical level, this study contributes by pointing to ways in which organizational environments and/or strategies can encourage employees to bring about innovative behavior and to lead themselves effectively in the workplace.

The results of the study indicate that SL operated as an intervening variable between EO and IWB. As hypothesized, SL fully mediates the relationship between EO and IWB. The result shows that the effect of EO on IWB is increased by developing SL skills. This result makes an important contribution to the literature. EO holds a prominent position by providing an environment to develop innovations in organizations. Taking steps only in boosting EO to enhance IWB is not enough; it is essential to place consideration on developing SL as well. The results of this study support that SL enhances IWB. It is also found that individuals who have strong SL are more likely to have high innovative behavior than individuals who have weak SL. Hence, SL notably helps individuals to develop IWB by providing individuals with self-management, self-motivation and self-influence on their own thoughts and/or behaviors. The development of innovative behaviors in organizations becomes easier with the SL skills that include the processes of self-influence and self-management, therefore organizations that seek out to facilitate IWB, need to recognize the importance of individuals’ SL skills.

De Jong and Den Hartog (2010) claim that despite the importance of IWB for organizational success, attempts to validate IWB measures have been scarce. Hence, in the present study, the reliability and validity of IWB scale were examined. The results of this study provide support for the validity and the reliability of IWB scale of De Jong and Den Hartog (2010) as an acceptable measure of IWB. Several scholars (e.g., Neck and Houghton 2006; Andressen et al. 2012) have also pointed out that that majority of SL research has been conceptual, with relatively few empirical studies in organizational setting. This study attempts to fill this gap in the literature.

The results of this study indicate that there is a significant difference between the SL scores of men and women, which is in agreement with the findings of Norris (2008). The women in the study scored higher than men. This situation can be the result of the extra responsibilities of women as a working mother. Another reason comes from the fact that women may be more collaborative, empowering and democratic in their leadership style (Eagly and Carli 2003). Furthermore, extant literature suggests several views about the differences in individual innovation exist among the various levels. Some scholars revealed that individual innovation decreases among employees as one moves down the hierarchy (Sebora et al. 1994; Fuller et al. 2006). Yet another view is that successful innovation activities in an organization require employee participation at all levels (Hartman et al. 1994; Ong et al. 2003; Wu et al. 2014). In agreement with the findings of Ong et al. (2003) and Wu et al. (2014), there is no difference between IWB among employees. The findings of the study also points out that there are no differences between participants’ perception of EO towards their organizations and SL in terms of positions. The reasons that the participants’ perception of EO towards their organizations, their IWB and SL are not affected by positions, can indicate that individuals do not understand the organizational environment distinctively and that individuals can be self-leaders and display innovative behavior regardless of their position. Another possible explanation for this might be because of the various innovation-based training programs that were implemented across the organization at all positions. It is therefore important for organizations to demystify individuals’ IWB and their SL skills at all positions, in order to improve the overall effectiveness of the organization.

The results of this study also have managerial implications. Due to the importance of the banking sector in developing countries, there is a need for banking managers to become efficient in managing innovative behaviors in order to support the constantly changing needs of customers and the rapidly changing market. If banking managers are interested in giving employees a sense of control over themselves, and building a sense of fostering innovativeness, proactiveness and taking risk, they can manage employees’ innovative behavior more effectively, and accordingly maximize employees’ IWB. The findings of this study also suggest critical implications in terms of both selecting and training employees and managers within banks. Banks should consider implementing SL selection standards and actively provide training programs in order to develop SL skills among employees and managers, which in turn promotes motivation to exhibit IWB.

## Limitations and future research directions

The results of the study should be considered in light of several limitations. Self-reported data from a single source may pose potential problems such as CMV. However, as discussed in the “Methods” section, the results of the study did not provide any indications of CMV. Although EO and SL have an important effect on IWB, the other individual and contextual factors affecting IWB can be identified. In the present study, the reason of collecting data from a banking industry is to minimize the cross-industry variations in work systems and job titles in selected organizations. Furthermore, the reason why the banking sector was chosen for the research is that in today’s world, technology is intensively used in banking sector with their diversified modern marketing techniques, products and processes. However, results may show difference for other sectors or industries. For future research, generalizations can be made in relation to these variables through different cultures, economies and sectors. Furthermore, future research might benefit from considering an intentional examination of gender on SL. Additionally, SL strategies and EO dimensions can be examined in future studies which can determine SL strategies and EO dimensions contributing to IWB.
